# Location and Movement of the Oxytocin Receptor Differ Between the Normal and Diseased Prostate

**DOI:** 10.3390/cancers17020182

**Published:** 2025-01-08

**Authors:** Maree Gould, Daniil Potapov, Helen Nicholson

**Affiliations:** 1 Department of Anatomy, University of Otago, P.O. Box 913, Dunedin 9054, New Zealand; helen.nicholson@otago.ac.nz; 2 Department of Physiology, University of Otago, P.O. Box 913, Dunedin 9054, New Zealand; daniil.potapov@otago.ac.nz

**Keywords:** oxytocin receptor, prostate, caveolae, single-molecule tracking, total internal reflection fluorescence microscopy

## Abstract

Background: In normal prostate cells, receptors for oxytocin (OT), a peptide involved in regulating prostate growth are sequestered within membrane microdomains called caveolae. During cancer progression, polymerase-transcript-release factor (PTRF) is downregulated, caveolae structures are lost and receptors move onto the cell membrane. This study investigated whether proteins responsible for caveolae formation were affected by the OT peptide, also, how OT treatment affected oxytocin receptor (OTR) movement within living cells. Methods: Normal human prostate epithelial cells (PrEC) expressing caveolin and PTRF and androgen-independent (PC3) cancer cells expressing caveolin but not PTRF were used. OTR, caveolin and PTRF expression was determined in human prostate tissue. Results: PTRF expression decreased in tissue alongside an increase in malignancy. Caveolin-1 expression was downregulated by OT treatment. Caveolin-2 was decreased by OT in PrEC cells but increased in PC3 cells. PTRF was decreased by OT in PrEC. TIRF microscopy showed OTR translocated from caveolae to caveolae in normal cells, whereas OTR moved without restraint in malignant cells, possibly stimulating signaling pathways. Conclusions: This study provides evidence for the ability of OT to regulate caveolin and PTRF expression. This study elucidates possible mechanisms by which cell receptors and caveolae proteins interact to enhance cell proliferation.

## Simple Summary

In normal prostate cells, receptors are sequestered within specialized cell membrane areas called caveolae. During cancer progression, a protein called polymerase transcript release factor (PTRF) that is essential for maintaining caveolae is decreased, causing the caveolae to disappear, and consequently, the receptors can move freely about the cell membrane. This study looked at whether oxytocin affects the proteins that form caveolae and how oxytocin can influence the movement of its receptor in living cells. In healthy prostate cells, oxytocin receptors move within caveolae, but in cancer cells, they move freely, potentially triggering growth signals. This study sheds light on how prostate cancer cells exploit the loss of the confinement of the receptors as these pathways could now promote uncontrolled growth. PTRF levels decrease as the malignancy progresses and could serve as a biomarker for prostate cancer diagnosis or progression. Additionally, a therapy that stabilizes or restores functional caveolae in cancer cells might inhibit cancer cell proliferation. Overall, this research highlights the potential of targeting membrane structure regulation, which could be a novel approach towards managing prostate cancer.

## 1. Introduction

In normal prostate cells, oxytocin (OT) is involved in the regulation of prostatic growth [1]. In these cells, the oxytocin receptor (OTR) is sequestered within the invaginations of the surface membrane that form caveolae [2]. Caveolae are formed from caveolin proteins, which include caveolin-1 and -2. Caveolin (cav) proteins normally co-localize and are critical for the formation of caveolae. These specialized stable structures are involved in cell signaling and trafficking [3].

Conversely, in prostate cancer cells, OT acts as a growth promoter [4]. In the presence of normal or reduced concentrations of androgens (as seen in androgen withdrawal therapy) OT stimulates extracellular signal-regulated kinase (ERK) phosphorylation and cell proliferation [5]. This proliferative effect can be mimicked by the disruption of lipid rafts in normal cells, indicating that a movement of OTR out of lipid rafts is involved [4]. In cancer progression, a loss of caveolae structures occurs [6], and the OTR moves out of caveolae onto the flat lipid rafts [7].

Polymerase I and transcript release factor (PTRF) is the molecule responsible for the drawstring structure of the caveolar sacks. PTRF is essential for the stabilization of caveolae at the cell membrane in normal cells; hence, caveolae are formed only when both caveolin and PTRF are present [8]. The prostate cancer cell line PC3 expresses high levels of caveolin-1 but does not express PTRF. In PC3 cells, PC3 cells stably expressing PTRF-GFP restored morphologically identifiable caveolae, reduced cell migration and lowered expression levels of matrix-degrading enzymes when compared to normal PC3 cells [9]. In DU145 prostate cancer cells, PTRF was downregulated using shRNA-mediated knockdown, leading to an increase in cell migration [10].

Both the stromal and epithelial cells in the normal human prostate produce oxytocin [1], and studies conducted both in vitro and in vivo have shown that these cell types express the receptors for OTR and the caveolin proteins [6]. There is evidence that plasma [11] and tissue [4] OT concentrations also decrease with the development of prostate cancer [1].

And yet, with the development of prostate cancer, there is an increase in the levels of cav-1 expression which promotes the further progression of metastatic cancer [12]. It is possible that the effects that OT has on cell proliferation may partly be due to changes in the expression of OTR and components of cell membrane, caveolins and PTRF. Is the effect due to the cancer itself or alterations in the cell membrane? An assortment of information exists on OT, OTR and caveolin regulation that requires clarification.

The aims of this study were three-fold: (1) determining whether the location of the caveolae-associated proteins was different across various states of disease, (2) determining whether the proteins responsible for the formation of caveolae are affected by treatment with differing concentrations of the peptide OT and (3) determining whether treatment with OT affects the movement of OTR in normal cells and malignant living human prostate cells.

## 2. Materials and Methods

### 2.1. Tissue Collection

Human prostate tissue was obtained from men undergoing routine surgical transurethral resection of the prostate (TURP). Ethical approval for the study was given by the Lower South Otago Regional Health and Disability Ethics Committee. Post-surgical prostate tissue was collected in cell culture media and kept on ice prior to use. Diagnosis for the cancer grade was made by a clinical pathologist. Tissue diagnoses included benign prostatic hyperplasia (BPH; *n* = 17), well-differentiated prostate cancer (Gleason grade 1–3; *n* = 9) or poorly differentiated prostate cancer (Gleason grade 4–5; *n* = 12). Normal prostate tissue samples (*n* = 3) were taken from the BPH tissue that displayed areas of normal morphology. 

### 2.2. Cell Culture

Normal human prostate epithelial cells (PrEC; CC-2555, Clonetics, BioWhittaker, Berks, UK lot.5F1289) were grown in accordance with manufacturer’s instructions in prostate epithelial basal medium (PrEBM, CC-3165) supplemented with bullet kit media (Clonetics, BioWhittaker, Berks, UK). Androgen-independent prostate cancer cells (PC3 cells (ATCC, Manassas, VA, USA, [13], Lot No. 22363) were received at passage 4 and were grown in F12/DMEM (Gibco 11320033, Invitrogen, Carlsbad, CA, USA), 10% fetal calf serum (FBS; Thermo Fisher Scientific, Auckland, New Zealand), 10,000 units Penicillin and 10 mg Streptomycin (Life Technologies, Auckland, New Zealand). All media were phenol red-free [14]. Cells were cultured at 37 °C in a humidified atmosphere of 5% CO_2_, and cell media were changed every 48 h until the cells were 80% confluent.

### 2.3. Double Fluorescent Immunocytochemistry

Immunocytochemistry (ICC) was used to investigate the localization and distribution of proteins in the prostate cell lines and prostate tissue. The specificity of the oxytocin receptor antibodies employed in this study was assessed through both inter-antibody comparisons and peptide-blocking studies [15]. For immunocytochemistry, cells were seeded at a density of 5 × 10^3^ for PrEC or 3.5 × 10^3^ for PC3 per mL to be at a comparable density at the time of the experiment. Cells were fixed in 4% paraformaldehyde for 5 min, or dewaxed sections of prostate tissue were blocked with 10% donkey serum (Sigma-Aldrich, Burlington, MA, USA) for 30 min. Samples were incubated with primary antibodies, either goat anti-OTR 1:50 (EB08990, Everest Biotech, Upper Heyford, UK), rabbit anti-cav-1 1:100 (ab2910; Abcam, UK), rabbit anti-cav-2 1:100 (ab2911; Abcam) or mouse anti-PTRF 1:40 (611258; Becton Dickinson), overnight at 4 °C in a humidity chamber. Negative control sections were incubated with mouse or rabbit IgG as appropriate. Secondary antibodies were either donkey anti-mouse labeled with AlexaFluor 555, donkey anti-rabbit AlexaFluor 488 or donkey anti-goat labeled with AlexaFluor 350 (Invitrogen, Whitefield, India). Slides were coverslipped using Vectashield mounting media (H-1000, Vector Laboratories, Newark, CA, USA). Slides were viewed under a BX-51 microscope with a fluorescent attachment (Olympus Corp, Westborough, MA, USA). The excitation/emission of AlexaFluor 350 was 343/440 nm, AlexaFluor 488 was 495/519 nm and AlexaFluor 555 was 555/565 nm. Images were captured and merged using the Spot imaging system (Diagnostic Instruments, Sterling Heights, MI, USA).

### 2.4. Western Blot Analysis

Western blots were performed to quantify protein expression, as previously described [15]. Cells were cultured in their respective media in the presence or absence of OT with the media changed every 24 h. Samples of 50 × 10^3^ cells per well were separated on a polyacrylamide gel (10%) and then transferred onto PVDF membranes that were blocked using Odyssey Blocking Buffer (LI-COR Biosciences GmbH, Bad Homburg vor der Höhe, Germany). The membranes were then probed with antibodies as described above using rabbit anti-α-tubulin 1:5000 as a loading control. The next day, the membranes were incubated with IRDye 680 goat anti-mouse IgG (926-32220; LI-COR) and 800 anti-rabbit IgG (926-32212; LI-COR). Blots were visualized using the Odyssey Infrared Imaging System (LI-COR), and integrated intensity data acquired. The antibody values were normalized to the loading control. Each experiment was repeated 3 times. 

### 2.5. In-Cell Western

Having determined that each antibody gave one clear single band on a Western blot, the In-Cell Western (ICW) method was used. For the ICW assay, 3 × 10^5^ PrEC cells or 1 × 10^5^ PC3 cells per well were seeded into 96-well plates. Treatments were added as described below. After 4 days, cells were fixed by the addition of 4% paraformaldehyde. Cell membranes were permeabilized with 0.1% Triton-X 100 and blocked in Odyssey Blocking Buffer (LI-COR) for 1 h. Cells were incubated at 4 °C with antibodies at the same concentrations as used for Western blots. The next day, cells were incubated with secondary antibodies as described above in Odyssey buffer for 1 h. Wells were washed, air-dried then scanned immediately on the Odyssey Infrared Imaging System (LI-COR) and integrated intensity data were acquired. The same wells were stained with Draq5 (AB108410; Sapphire Bioscience, Auckland, New Zealand) as a control that normalizes antibody signals to cell number. Each experiment was performed in triplicate on three separate occasions.

### 2.6. Treatments

Cells were cultured in the presence of media alone or in the presence of OT at physiological (10 nmol·L^−1^) or at reduced concentrations (0.1 nmol·L^−1^) similar to those found in vivo in prostate cancer [1].

### 2.7. Single-Molecule TIRF Microscopy

The detection of the trajectory of a single molecule allows the visualization of real-time movement of the OTR in living cells. Total internal reflection fluorescence (TIRF) microscopy is based on the principle that the intensity of the evanescent wave exponentially decays and only selectively excites fluorescent molecules less than a hundred nanometers from the plasma membrane. For TIRF microscopy, cells were seeded into 24-well plates containing 18 mm glass coverslips at a density of 5 × 10^3^ for PrEC or 3.5 × 10^3^ for PC3 in a final volume of 1 mL of their respective culture medium and allowed to adhere overnight at 37 °C/5% CO_2_ humidified incubator. Cells were incubated for 15 min with primary antibody goat anti-OTR 1:100 (N-19; Santa Cruz Biotech, Dallas, TX, USA). OTR-N19 antibody targeting the extracellular N-terminal portion of the OTR was used because these cells were alive and not permeabilized. Negative controls using an equivalent concentration of goat IgG were always used. After washing, cells were incubated in biotin-conjugated F(ab’)_2_ donkey anti-goat IgG (705-066-147; Jackson, UK) for 15 min and then incubated with streptavidin-conjugated Qdots (Q10121MP; Invitrogen, Whitefield, India) for 3 min before washing twice in media. The Qdots have a peak emission wavelength of 655 nm. Coverslips containing the Qdot-conjugated normal and cancerous cells were placed onto the heated conical holder containing respective color-free media without FBS or OT. The holder was positioned below an inverted TIRF Olympus IX81 microscope, which uses a Spectral LMM ND401 laser with a 655 filter (Olympus Corp, Shinjuku City, Tokyo, Japan), and the samples were observed using a high numerical aperture objective. 

A 1 mL syringe containing media containing 100 nmol·L^−1^ OT or media alone was positioned into a Microinject diffusion pump (HT life science, West Lafayette, IN, USA). Baseline values were measured before application. After 2 min of recording treatments using media only, and then media plus OT (final 10 nmol·L^−1^ concentration within the chamber) were added, and the movement of each OTR was recorded. A single series of images was captured for each coverslip, with at least 3 independent experiments for each treatment. Each series consisted of approximately 8000 frames, with a duration of 42 ms each, totaling 6 min of recording.

Qdots that were bound were easily identified due to their bright fluorescent blinking as they transitioned between the fluorescent (ionized) and non-fluorescent (neutralized) states. Each coverslip could be scanned for up to 1 hour without any noticeable detrimental effects on the cells. The image series were captured using MetaMorph software 7.7.2.0 (Molecular Devices Inc., San Jose, CA, USA).

### 2.8. Calculation of the Mobility Parameters of a Single Molecule

The equation of mean squared displacement (MSD) of the diffusing particles was obtained to distinguish between normal and anomalous diffusion; this was done by performing a temporal average over one single trajectory [16]. From there, an assembled trajectory composed from a single Qdot’s image sequences was broken into shorter time averages or displacements. These were considered as independent variables on a sliding scale, and the variance of this distribution was determined as MSD. Parameters taken from each trajectory were determined using a formula taking into account the blinking occurrences of the Qdots [17]. The displacement calculation provided data for an intertrajectory comparison of each of the treatment groups. Regions of non-random behavior are expressed as L probability. If a segment of movement represents random Brownian motion, then L = 0. Higher L values indicate greater non-random confinement. The greater the tendency for non-random confinement, the higher the value of L probability will be. L was plotted over time for each segment to show the trajectory profile. Non-random confinement was determined as being the period of time the protein remained immobile for a duration considerably longer than could be explained by Brownian motion [18]. Mobile versus immobile periods (IPs) were also determined [19]. Data analysis was performed as defined by Kwakowsky et al. [20].

### 2.9. Immunogold Staining for Transmission Electron Microscopy

Immunogold staining is a subtle balance between good morphology via fixation and immunogold staining providing a decent signal. Identification of caveolae in the cells can be established by antibodies that identify caveolin proteins. Transmission electron microscopy (TEM) was used as caveolae are too minute to be identified using a light microscope. Once the caveolae structure was located, then the OTR localization could be established. 

Wells containing cells grown on Thermanox coverslips were incubated in media without FBS at 37 °C in a 5% CO_2_ atmosphere in a humidified incubator for 10 min before fixation. Then, a portion of the tissue culture medium was withdrawn, and an equal quantity of pre-warmed 2% glutaraldehyde/2% paraformaldehyde in 0.1 M phosphate buffer (PB) containing 2 mM CaCl_2_ was added and incubated for 1 min. After the fixative was removed, it was replaced with fresh fixative and incubated for 30 min at 37 °C. The samples were washed in 0.1 M PB and then washed with 0.1% sodium borohydride in 0.1 M PB for 10 min to inactivate residual aldehyde groups, followed by several PB washes. Permeabilization of the cells was performed using 0.05% Triton in 0.1 M PB for 10 min, and then the cells were blocked using a high-molecular-weight protein for 15 min and washed with incubation buffer (Aurion, Wageningen, The Netherlands). Primary antibody rabbit anti-cav-1 or goat anti-OTR (N19) or PBS as a negative control was used in incubation buffer in a humidified chamber overnight at 4 °C. 

On day 2, samples were incubated with secondary antibodies of either donkey anti-goat (0800.333) or anti-rabbit (0800.311; Aurion, Singapore) with ultrasmall gold conjugate 1:100 in incubation buffer overnight at 4 °C. 

On day 3, samples were washed with incubation buffer and ddH_2_O with post-fixation in 2% glutaraldehyde in PB for 5 min. After washing again with ddH_2_O, samples were silver-enhanced using fresh R-Gent solution (500.033, Aurion, Singapore) until brown for 1 h. Samples were washed thoroughly with ddH_2_O and then post-fixed with 0.5% OsO_4_ in ddH_2_O for 15 min. Samples were washed with ddH_2_O and dehydrated through graded ethanol into 100% ethanol before infiltration with propylene oxide (PO) and then 1:1 PO/Agar 100 (Agar Scientific, Essex, UK) for 5 min. Finally, cells were infiltrated with resin for 4 × 30 min. The processed coverslip was placed upon a BEEM capsule filled with resin and polymerized for 24 h at 60 °C. The coverslip was removed using minimal heat, and embedded cells were identified by staining with Methylene blue–azure II stain, and a small resin block was cut out. Blocks were orientated 180° so that vertical sections could be taken through the cells, embedded back into fresh resin and polymerized for 48 h at 60 °C. Ultrathin (80 nm) sections were cut on a Reichert FCS microtome using a Diatome diamond knife. Contrast was given using 1% buffered uranyl acetate and Reynolds lead citrate. Grids were observed using a Phillips CM100 TEM (Philips, Eindhoven, The Netherlands) at 80 kv accelerating voltage whilst the SIS Mega III acquired images.

### 2.10. Statistical Analysis

Data are given as mean ± standard error of the mean (SEM) of triplicate experiments. Student’s *t*-test compared the two cell types with a significance level set at *p* < 0.05. A one-way analysis of variance (ANOVA) with a post hoc Bonferroni correction test was used to determine the significance of differences across all groups. Trajectory and displacement calculations were determined using a custom-made Matrix Laboratory (MatLab) function (MathWorks, Carlsbad, CA, USA). All statistical analyses were performed using GraphPad Prism 4.0 (GraphPad Software Inc., San Diego, CA, USA).

## 3. Results

### 3.1. Immunocyto- and Histochemistry

#### 3.1.1. Immunofluorescence

##### Double Immunofluorescence for PTRF and OTR

**Figure 1:**
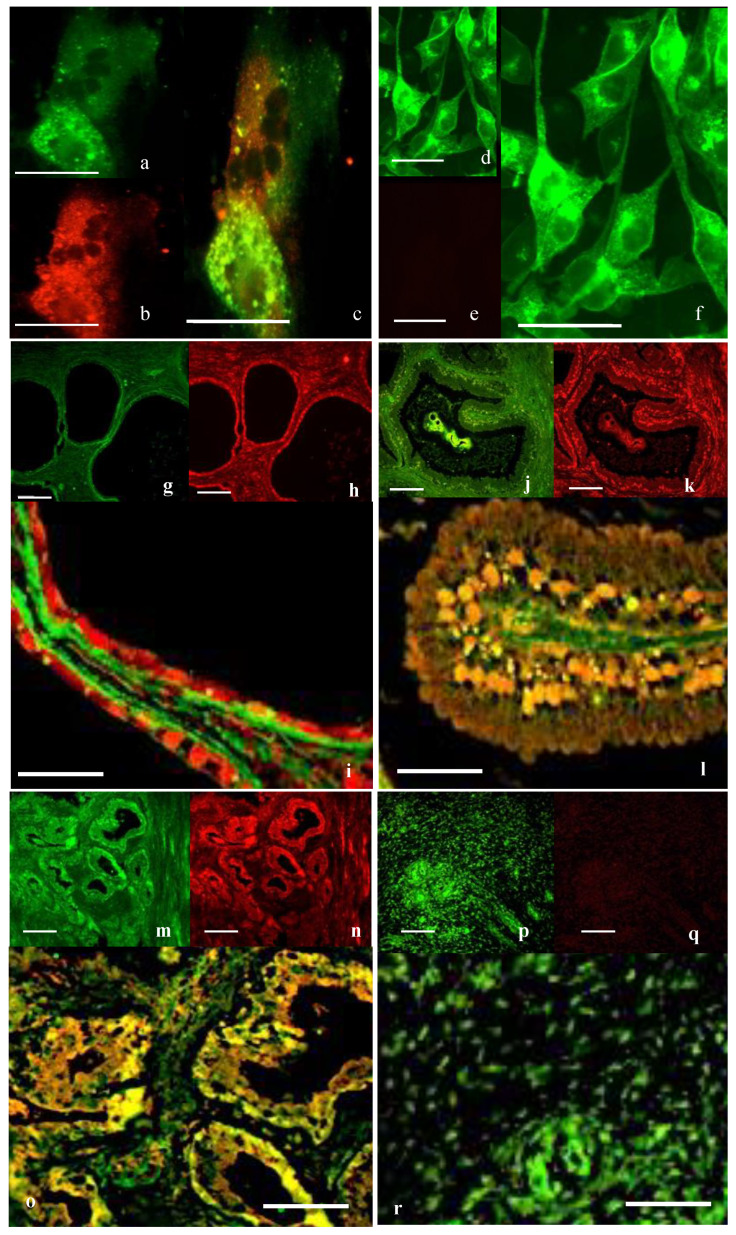
Double immunofluorescence for OTR and PTRF in cells and tissue. OTR (in green, (**a**,**d**,**g**,**j**,**m**,**p**)) and PTRF (in red, (**b**,**e**,**h**,**k**,**n**,**q**)) with co-localization (**c**,**f**,**i**,**l**,**o**,**r**) seen as yellow. In PrEC (**a**–**c**), OTR and PTRF were expressed. PC3 cells (**d**–**f**) showed no expression for PTRF but strongly expressed OTR. Very little co-localization was observed in normal prostate tissue (**g**–**i**); some co-localization was seen in BPH (**j**–**l**) which increased in well-differentiated cancerous tissue (**m**–**o**). In poorly differentiated cancerous tissue (**p**–**r**), PTRF immunoreactivity decreased, whereas OTR was still expressed. Bars = 50 µm. Oxytocin receptor (OTR), polymerase I and transcript release factor (PTRF), prostate epithelial cell line (PrEC), prostate cancer cell line (PC3), benign prostatic hyperplasia (BPH).

Strong co-localization for OTR and for PTRF was seen on the surface in PrEC cells (Figure 1a–c). Immunostaining of OTR was seen in PC3 cells, but no immunofluorescence for PTRF was detected (Figure 1d–f).

Double immunofluorescence for OTR and PTRF showed that co-localization in the epithelial or stromal cells in normal tissue was not seen (Figure 1g–i). In BPH tissue, definitive co-localization was seen in the nuclei and weaker co-localization in the cell membrane of stromal and epithelial cells (Figure 1j–l). In well-differentiated cancerous tissue (Figure 1m–o), a lesser epithelial expression of PTRF was seen, which was lost altogether in the poorly differentiated tissue (Figure 1p–r).

##### Double Immunofluorescence for Caveolin-1 and OTR

**Figure 2:**
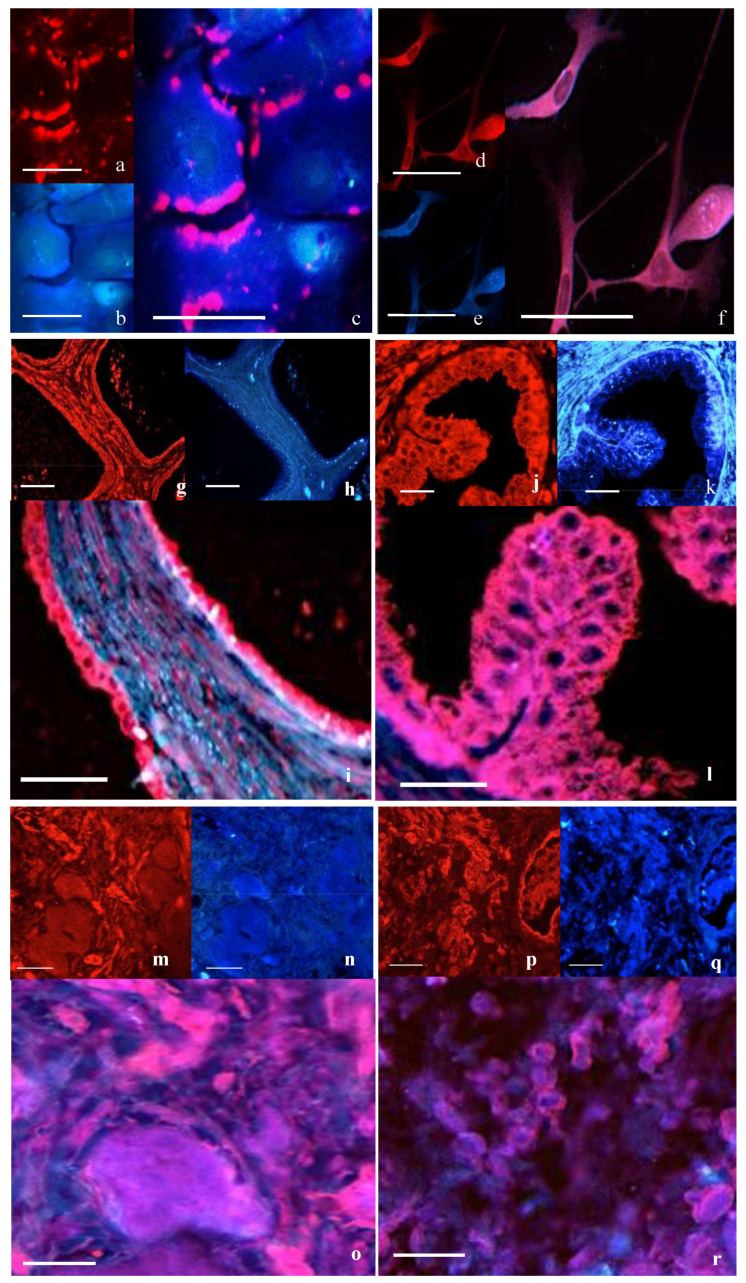
Double immunofluorescence for cav-1 and OTR in cells and tissue. Cav-1 (in red, (**a**,**d**,**g**,**j**,**m**,**p**)) and OTR (in blue, (**b**,**e**,**h**,**k**,**n**,**q**)) with co-localization (**c**,**f**,**i**,**l**,**o**,**r**) seen as purple. In PrEC (**a**–**c**), cav-1 was strongly localized around the cell margin. PC3 cells (**d**–**f**) showed co-localization with cav-1 and OTR. Co-localization was observed in normal prostate tissue (**g**–**i**) and in BPH (**j**–**l**). Co-localization was also seen in well-differentiated (**m**–**o**) and in poorly differentiated cancerous tissue (**p**–**r**). Bars = 50 µm.

Caveolin-1 immunoreactivity was seen clumped along the cell margins in the PrEC cells (Figure 2a–c). Immunoreactivity for cav-1 was more uniformly distributed on the cell membrane of PC3 cells and co-localized with OTR (Figure 2d–f).

Double immunofluorescence of OTR and cav-1 in prostate tissue epithelial cells showed strong co-localization, whilst stromal cells showed some co-localization in the normal (Figure 2g–i) and BPH tissue (Figure 2j–l). In the well-differentiated (Figure 2m–o) and poorly differentiated cancerous tissue (Figure 2p–r), co-localization was seen throughout the tissue.

##### Double Immunofluorescence for Caveolin-2 and OTR

**Figure 3:**
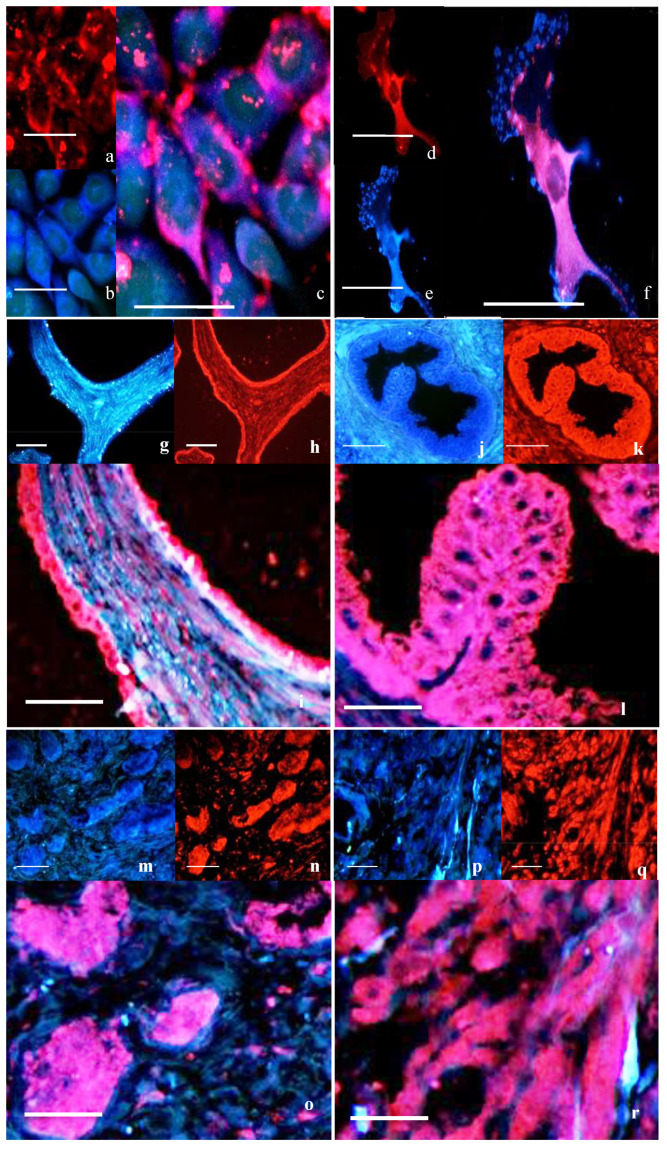
Double immunofluorescence for cav-2 and OTR in cells and tissue. Cav-2 (in red, (**a**,**d**,**h**,**k**,**n**,**q**)) and OTR (in blue, (**b**,**e**,**g**,**j**,**m**,**p**)) with co-localization (**c**,**f**,**i**,**l**,**o**,**r**) seen as purple. In PrEC (**a**–**c**), cav-2 was strongly localized around the cell margin. PC3 cells (**d**–**f**) showed co-localization with cav-2 and OTR. Co-localization was observed in normal prostate tissue (**g**–**i**), in BPH (**j**–**l**) and in well-differentiated (**m**–**o**) and poorly differentiated cancerous tissue (**p**–**r**). Bars = 50 µm.

In normal epithelial cells (Figure 3a–c), patchy co-localization immunoreactivity for cav-2 was seen along the cell margins which co-localized with OTR. Co-localization was seen between cav-2 and OTR in the cell membrane of the PC3 cells along with distinct regions of OTR staining (Figure 3d–f).

Double immunofluorescence for OTR and cav-2 in prostate tissue showed a similar staining pattern to cav-1 with strong co-localization in the epithelial cells and less co-localization in stromal cells in normal (Figure 3g–i) and BPH tissue (Figure 3j–l). In well-differentiated (Figure 3m–o) and poorly differentiated cancerous tissue (Figure 3p–r) some co-localization was seen, predominantly in the epithelial cells.

### 3.2. Ultrastructural Analysis

#### Results

Ultrastructural analysis using immunogold determined the localization of both caveolin and OTR in cell lines. Identification of caveolae in the cells was confirmed by the use of antibodies raised against caveolin protein. These structures are easily identifiable in the normal cells, and as previously determined, caveolae were lost in the malignant cell lines.

**Figure 4:**
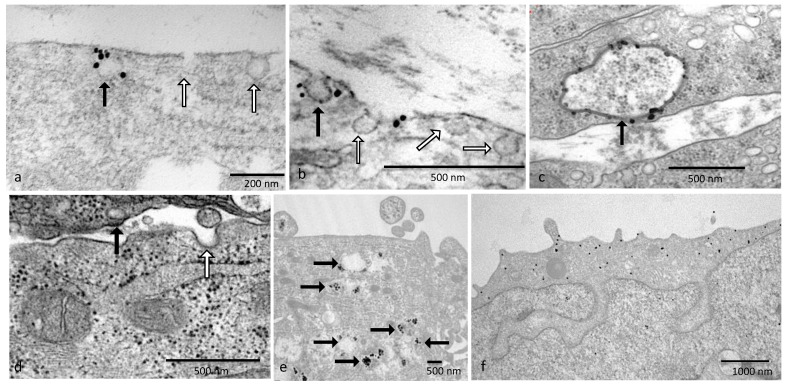
Electron micrographs of oxytocin receptor and caveolin immunogold staining. Electron micrograph of (**a**–**c**) caveolae vesicles from prostate epithelial cells stained with caveolin-1 and immunogold particles (seen as black beads). (**d**) Clathrin pits did not stain with antibodies to cav-1 or cav-2. Black arrows indicate caveolae. White arrows indicate clathrin pits. (**e**) OTR-N19 was associated with caveolae in normal cells (**f**) and more dispersed in malignant cells. Micron markers are noted on images.

Cell surface immunoreactivity for caveolin was seen in normal prostate cells grown in vitro (Figure 4a–c). Malignant PC3 cells showed strong immunoreactivity for OTR on the cell membrane and within the cells (Figure 4e–f).

### 3.3. Protein Expression of Caveolae Proteins

#### 3.3.1. Results for Western Blot Analysis of Caveolin and PTRF in Normal and Cancer Cell Lines

##### The Effects of OT on Caveolin-1, -2 and PTRF Protein Expression in PrEC and PC3 Cells at 4 Days

**Figure 5:**
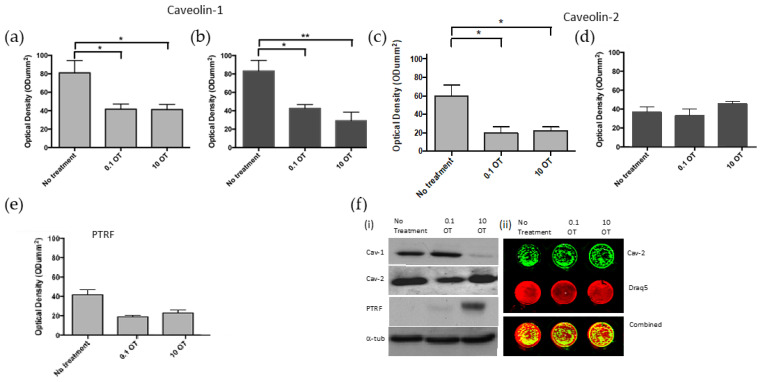
The effects of OT on caveolin-1, -2 and PTRF protein expression in normal human prostate epithelial (PrEC) cells and malignant human prostate (PC3) cells at 4 days. Protein expression of caveolin-associated proteins in normal PrEC (pale grey; (**a**,**c**,**e**)) or PC3 cells (dark grey; (**b**,**d**)). Prostate cells treated with OT at reduced or physiological levels. Caveolin-1 at 22 kDa in (**a**) normal and (**b**) malignant cells and caveolin-2 at 21 kDa in (**c**) normal and (**d**) malignant cells at 4 days. PTRF at 43 kDa in (**e**) normal cells at 4 days. All blots were run on a single gel. (**f**(**i**)) Western blots of caveolin-1, -2 and PTRF along with a representative depiction of the (**f**(**ii**)) In-Cell Western technique, performed using the PC3 cell line, normalized to Draq5 as a control, ensuring that antibody signals are adjusted based on cell number. Bars = mean + SEM. *n* = 3. * *p* < 0.05, ** *p* < 0.01.

There was a significant decrease in caveolin expression in normal cells treated with low or physiological levels of OT compared to the no-treatment control (ANOVA, *p* = 0.032; Figure 5a).

At 4 days, a significant decrease in cav-1 expression was seen in PC3 cells treated with low or physiological levels of OT compared to the no-treatment control (ANOVA, *p* = 0.006; Figure 5b).

Normal cells showed a significant decrease in cav-2 expression when treated with 0.1 and 10 nmol·L^−1^ OT (ANOVA, *p* = 0.012; Figure 5c). Malignant cells showed no significant changes in cav-2 expression when treated with OT (ANOVA, *p* = 0.26; Figure 5d).

Normal cells treated for 4 days showed a significant decrease in PTRF expression when treated with OT alone (ANOVA, *p* = 0.0037; Figure 5e). Malignant PC3 cells that do not express PTRF were not included.

#### 3.3.2. Results for Western Blot Analysis of Caveolin and PTRF in BPH and Cancerous Tissue

**Figure 6:**
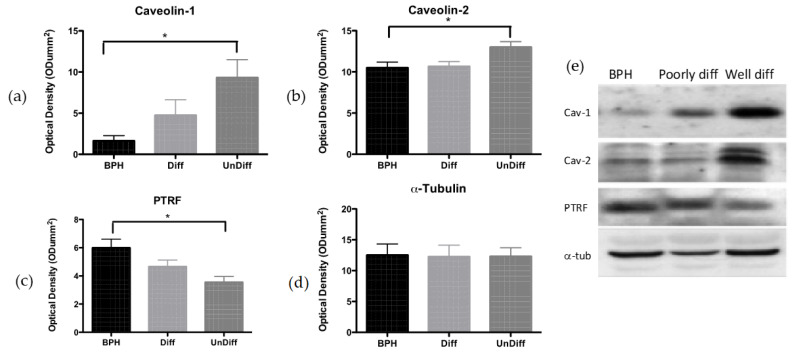
Western blot analysis of caveolin and PTRF in BPH and cancerous tissue. (**a**) Analysis of caveolin-1 at 22 kDa in human prostate tissue. *n* = 3. (**b**) Western blot analysis of caveolin-2 at 21 kDa in tissue. *n* = 6, 7, 5. (**c**) Western blot for PTRF at 43 kDa. *n* = 10, 7, 7. (**d**) *α*-Tubulin at 50 kDa was used as a loading control *n* = 8. BPH = benign hyperplastic prostate tissue; Diff = well-differentiated prostate cancerous tissue; Undiff = poorly (or undifferentiated) prostate cancerous tissue. (**e**) Representative blots. Bars signify mean + S.E.M. * *p* < 0.05.

Caveolin 1 expression was increased in the undifferentiated malignant tissue compared to the BPH tissue (Figure 6a; *p* = 0.0315). Similar results were seen with cav-2 (Figure 6b; *p* = 0.0316). With the loss of the caveolae structures, it was of no surprise that PTRF was significantly decreased in undifferentiated tissue (Figure 6c; *p* = 0.014). *α*-Tubulin was used as the loading control (Figure 6d; *p* = 0.99).

### 3.4. TIRF Cell Membrane Analysis

#### TIRF Results

Real-time imaging of the OTR with Qdot labeling allowed tracking of the receptor as it moved across the cell membrane of living cells. The identification of OTR in the cells was determined by the blinking/non-blinking signal. Cells were initially observed without any addition to the chamber to set baseline measurements. Although the addition of media alone caused turbulence, the tracked movement of the receptor remained unaffected.

**Figure 7:**
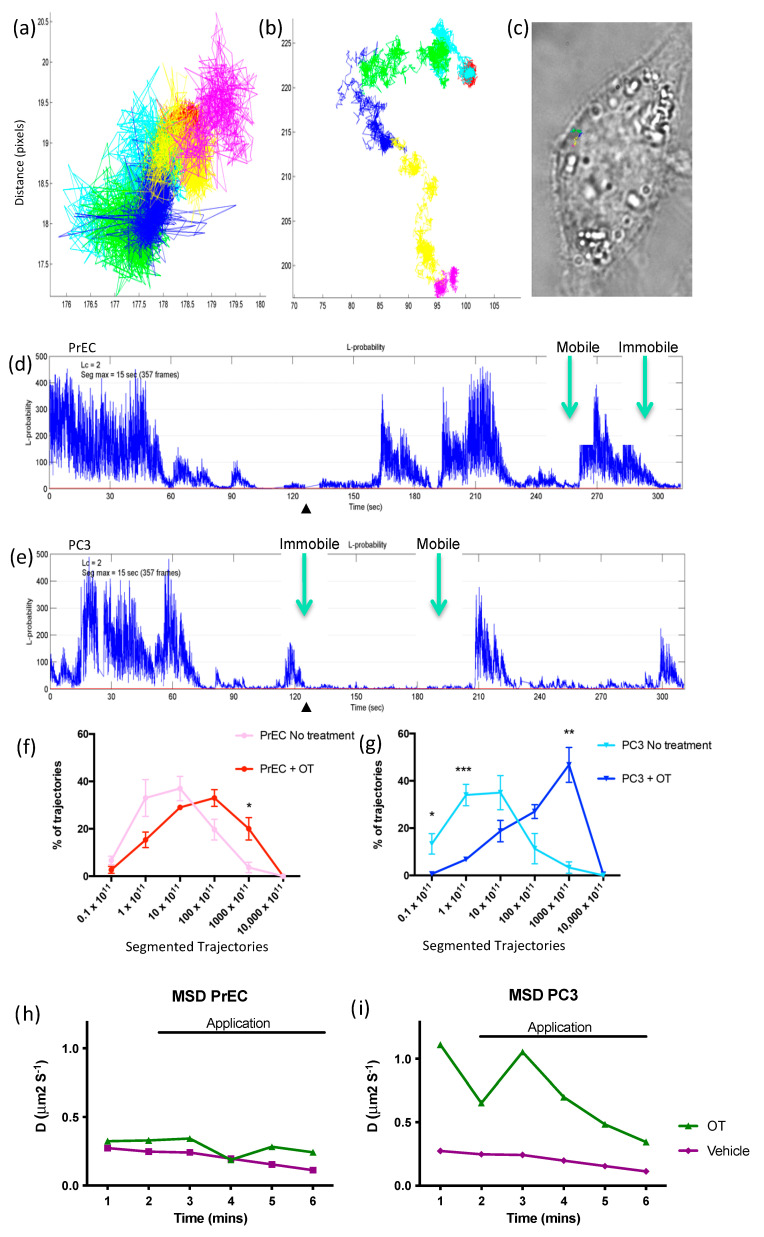
TIRF microscopy. Representative single-molecule tracks of the oxytocin receptor (OTR) in (**a**) normal human prostate epithelial (PrEC) cells and (**b**) malignant human prostate (PC3) cells following treatment with OT. Each color represents one minute of trajectory with one molecule over a total time of 6 min. (**c**) OTR tract to scale on a malignant cell surface. Line plots of fluorescence intensity with time for (**d**) PrEC and (**e**) PC3 cells showing the mobile and immobile phases with the addition of OT at the arrowhead. Green arrows indicate the mobile and immobile periods. The percentage of trajectories in (**f**) normal and (**g**) malignant cells treated and not treated with OT. The variance of these segments was calculated as the mean square displacement (MSD) over time for (**h**) PrEC and (**i**) PC3 cells. *n* = 3. * *p* < 0.05, ** *p* < 0.01, *** *p* <0.001.

A single molecule of OTR tracked across the surface of a living prostate cell showed a distinctive difference in movement between the two cell types after the addition of OT. In both cell types, the OTR remained motionless until stimulated with OT. In normal cells, a responsive movement was seen with the addition of OT, but the movement remained confined within a restricted area (Figure 7a). In the malignant cells, the responsive movement was greater with the addition of OT, as the OTR moved haphazardly across large areas of the cell membrane without restraint (Figure 7b,c).

If movement was due to random Brownian motion, then L would equal 0, and the greater the tendency for non-random confinement, the higher the value of L. In other words, if the OTR has movement, then the L will be lower; conversely, confinement of the OTR molecule means the L will be increased. Comparing the two cell types showed that there were significant differences in movement of the OTR. In Figure 7d, for PrEC treated with OT, movement was more confined and ceased for extended periods of time, whereas in Figure 7e, in PC3 cells, movement was more dynamic and OTR only stopped transiently. This difference in L highlights disparities in the OTR response to OT treatment between normal prostate epithelial cells and cancerous prostate cells.

When an intensity trajectory was plotted for a single molecule of OTR within a cell, distinct periods of immobility (IP) were observed. The total size of the movement within the immobile time frame is shown with approximately 98 IPs tracked with a representative trace of a trajectory of *a* single OTR. Displacement parameters taken from each trajectory in the PrEC cells (Figure 7f) showed that when PrEC were untreated, the OTR was immobile and with the application of OT alone, there was a slight rise in displacement compared to the untreated sample. When displacement trajectories from the PC3 cells were combined (Figure 7g), the addition of OT resulted in a distinct peak in displacement at the time of application compared to the cells with no treatment, as evidenced by the shift to the right in the line graph.

Displacement parameters over time show that in normal cells, the application of OT at 1 minute only showed a very slight increase in displacement (Figure 7h). In the malignant cells, the application of OT provided a much greater increase in displacement (Figure 7i). In viewing media only, no difference is seen before and after application. The displacement analysis provided the displacement coefficient of a single molecule undertaking confined non-Brownian motion in the presence of the OT ligand. When both cell types were stimulated with OT, there was a distinct period of immobility as the OTR remained confined within the caveolae, and then the receptor moved again until the next period of immobility.

## 4. Discussion

This study provides evidence that in normal/BPH tissue and in PrEC cells, OTR co-localizes with caveolin and PTRF. Furthermore, ultrastructural analysis indicates that in normal prostate cells, OTR is located within the caveolae, but this was not observed in cancer cells. OT downregulates the formation of caveolae by downregulating the expression of key caveolae proteins while the OTR is sequestered and more controlled within caveolae. While OT is considered a putative tumor suppressor, its ability to act as such raises questions about whether the freeing of OTR from prostate cancer cells that lack caveolae is associated with an increase in growth signaling where OTR is more available to interact with ligands and potentially activate growth signaling pathways.

Prostate cancer representative PC3 cells express abundant caveolin proteins but no PTRF. In a previous study, we have shown that in prostate cancer, the numbers of caveolae significantly decrease due to the loss of PTRF expression [6]. In this study, there was a striking interrelation between PTRF and caveolins with respect to OTR. In normal human prostate tissue, the immunostaining for OTR and cavs/PTRF was discrete and individual, but as the disease advanced to a more malignant state, the extent of co-localization increased, until PTRF was no longer seen in the undifferentiated tissue, but interestingly, the caveolin proteins remained. For caveolae to form, then PTRF must be expressed, As the caveolae where the OTR resides are lost, then the areas of co-localization become less defined and more dispersed. The assumption is that the previously sequestered OTR moves onto the flat cell membrane rather than within the caveolae. The fact that similar changes are seen both in cells and in cancerous tissue suggests that in vitro findings reflect the in vivo situation.

Ultrastructural analysis using immunogold determined the localization of both caveolin and OTR in cell lines. These structures are easily structurally identifiable in the normal cells, and as previously discussed, caveolae are no longer present in the malignant cell lines. Guzzi et al., in 2002, used fractionation procedures to show that only between 1 and 15% of total OTRs were partitioned within caveolae in Madin–Darby canine kidney (MDCK) cells, whereas the majority of OTR-GFP fusion proteins were retrieved from the lower fractions that held the greater part of membrane-associated proteins [5], providing supporting evidence that OTR was sited upon the cell membrane surface [4]. Overall, it may be the distribution of the receptor between caveolae and lipid rafts along with the percentage of OTRs sequestered within the caveolae that may be of utmost importance.

Immunopositivity for PTRF was detected in epithelial cells and tissues from patients with BPH. In cancerous prostate tissue, a reduction in the expression of PTRF transpired not only in the epithelial cells but also in the stromal cells, suggesting that malignant epithelial cells may modify expression patterns in the surrounding stromal tissue [13]. Notably, BPH tissue showed abundant stromal cav-1 immunostaining, whereas CaP showed an absence of stromal cav-1 directly correlating with CaP progression, as tumor metastases have been shown as negative for stromal cav-1 immunostaining [14]. 

There is growing evidence to suggest that caveolin protein expression not only provides the structural component of caveolae, but also, in prostate cancer, operates as a tumor suppressor [15]. Urinary tract tumors, including prostate but also kidney [16] and bladder cancers [15], show increased levels of cav-1. In this study, cav-1 expression was decreased in both normal and cancer cells with the addition of OT. Furthermore, this decrease was seen in cav-1 protein expression, but not in malignant cells expressing cav-2. Caveolin-1 has been shown to decrease apoptosis [17], and it is unclear whether cav-2 has a similar effect. 

In patients with breast cancer, both cav-1 and cav-2 are linked to basal-like tumors and those that lack positive steroid hormone receptor expression (triple-negative phenotype). Univariate analyses reveal that only cav-2 has a prognostic significance for breast-cancer-specific survival [18]. Additionally, PTRF and Cav-1 interact with insulin-like growth factor-I receptor (IGF-IR), regulating its internalization in breast cancer cells that are known to express high levels of IGF-IR [19]. We showed earlier that cav-2 was significantly increased in prostate cancer compared to cav-1 [2,6]. An increase in cav-2 expression has also been linked with cancer progression in aggressive forms of esophageal [20] and pancreatic cancer [21]. Further studies are recommended to determine the roles that cav-1 and -2 play in CaP development.

In the normal prostate cells, cav-1 and -2 expression was observed around the cell margins. Other researchers have also shown the distribution of cav-1 in confluent normal NIH 3T3 cells was also localized around the cell margins [22]. In the cancer cells in this study, localization to the cell margin was absent. Cell-to-cell contact is lost in the progression of malignancy; therefore, caveolin expression may be important in normal cells for mediating cell-to-cell contact inhibition, possibly by negatively regulating the activation of the Ras-p42/44 MAP kinase cascade [23]. In normal prostate cells, co-localization was observed at the cell margins where cav-1 was expressed, while OTR was expressed in the remainder of the cell. In malignant cells and cancerous tissues, OTR strongly co-localized with the caveolin proteins, indicating that despite the loss of caveolae structures, OTR remains associated with caveolin proteins. This study provides evidence that the location of the OTR within the cell membrane influences the outcome of agonist binding. In the malignant cells, there was significant co-localization between cav-1 and -2 with OTR. With the loss of the caveolae structures, OTR (and other GPCRs) would have to translocate to the cell membrane potentially under the stimulation from OT. In breast cancer, tumor cells invade by remodeling the extracellular matrix, with the cell invadosomes interacting with caveolae clusters to repurpose plasma membrane subdomains, thereby supporting stromal matrix remodeling and facilitating tumor invasion [24]. 

Caveolin proteins were expressed in BPH tissue, and the expression was seen to increase; conversely, PTRF expression decreased alongside the progression of CaP. PTRF is essential for caveolae formation, and its reduced expression correlates with the absence of caveolae structures [25]. Furthermore, these results show that in prostate cancer, cav-2 was not associated with caveolae as while PTRF and caveolae were lost in PC3 and CaP tissue, cav-2 expression remained unchanged. Although changes in cav-1 have been documented previously in CaP [26], our data provide the first evidence that cav-2 may also undergo changes, suggesting that upregulation of cav-2 could be more pronounced than cav-1. 

One of the well-established roles of OT in normal prostate epithelial cells is the inhibition of cell proliferation [4]. In normal prostate epithelial cells, OT downregulated caveolin-1, -2 and PTRF expression. Regulation of caveolin expression by OT has not been shown previously. This downregulation of caveolae proteins by OT may be a regulatory mechanism critical for control over the proliferative effect of OT on normal cells. The downregulation of PTRF may also be involved in the inhibitory effect of OT on normal prostate cell proliferation. The consequential decrease in the number of caveolae may be a regulatory method required to control cell signaling. We have previously shown that in normal cells, treatments with OT increased ERK1/2 expression despite a lack of effect on cell proliferation. p-ERK is known to play a role in cell proliferation; thus, we anticipated a change in p-ERK expression in cancer cells, but the prostate cancer cells did not show activation of ERK phosphorylation [2]. This observation aligns with findings that poorly differentiated prostate cancers exhibit lower levels of p-ERK compared to well-differentiated cancers [27]. Activation of the ERK pathway has been reported in both the human RWPE prostate epithelial cell line and the WPMY prostate stromal cell line. Additionally, OT treatment in rodents has been shown to promote prostate enlargement via the MEK/ERK/RSK pathway [28].

These data demonstrate the effects of treating normal and malignant prostate cell lines with physiological and reduced levels of OT and the consequential changing expression of PTRF and caveolin proteins. These results show that OT treatment of PrEC and PC3 cells downregulated PTRF and caveolin proteins -1 and -2. OTR was seen to initially co-localize in discrete areas with caveolae markers in the normal and BPH tissue, but co-localization decreased as the cancer progressed. This was supported by data showing that the receptor differs in its movement on the normal and malignant cell membranes. 

In this study, in malignant cells, treatment with OT resulted in the movement of OTR out of lipid rafts and downregulated expression of cav-1. Caveolin expression may be regulated not only by OT but also by the other receptors that interact with caveolin proteins. Future work will explore the signaling proteins associated with these interactions. 

Tracking of a single molecule of OTR across the surface of living prostate cells revealed a disparity in movement and distance traveled between the two cell types. Immobilization periods (IPs) refer to distinct time intervals during which a molecule, such as a receptor, exhibits limited or no movement within a cellular environment where it may become temporarily tethered to cell structures in a manner that restricts its mobility [29]. In normal cells, the addition of OT prompted a responsive movement that remained confined within a limited area. In the malignant cells, the responsive movement was more pronounced, with OTR moving erratically across large areas of the cell membrane without constraint. In PC3 cells, OT stimulates cell proliferation [4], and this may be due to the changing location of the OTR stimulating different cell signaling pathways. The retention of OTR on the cell membrane allows activation of the signaling cascades, which regulate various cellular processes. The observed confinement occurring within regions of 100–300 nm^3^ in diameter was attributed to interactions between OTR and either caveolae or lipid rafts, highlighting a significant amount of temporary confinement in the protein trajectories. Similar observations have reported that in dermal fibroblasts, the LDL receptors on the cell surface are mobile and move distances of 0.5–1 μm, comparable to the average spacing between coated pits, and those receptors in the coated pits also displayed detectable mobility [30].

Further investigation needs to be performed on the prostate and the role that the caveolin and its associated proteins play in cancer progression and metastasis.

## 5. Conclusions

Caveolin proteins interact with PTRF to form stable caveolae structures. Treatment of normal cells with OT resulted in decreased caveolae proteins, namely cav-1, cav-2 and PTRF, possibly as a regulatory method for controlling cell proliferation. In normal cells, OTR moved from caveolae to caveolae, but with the loss of these structures, the OTR moved unrestrained. In prostate cancer, PTRF expression was lost, and the levels of OT became diminished, removing this regulation over caveolin alongside a consequential upregulation of cav-1 and particularly cav-2 expression. In malignant cells, the OT treatment decreased cav-1 expression, indicating that oxytocin may be involved in the regulation of PTRF and caveolin in the diseased prostate.

The loss of OT and PTRF expression in the malignant prostate resulted in increased caveolin proteins that may enhance the progression of prostate cancer. Potentially, PTRF has the potential for use as a biomarker in the differentiation of cancer from benign disease.

## Data Availability

Dataset available on request from the authors.
